# *QuickStats:* Percentage* of Adults Aged ≥18 Years with Diagnosed Diabetes,**^†^** by Disability Status**^§^** and Age Group — National Health Interview Survey,**^¶^** United States, 2020

**DOI:** 10.15585/mmwr.mm7104a6

**Published:** 2022-01-28

**Figure:**
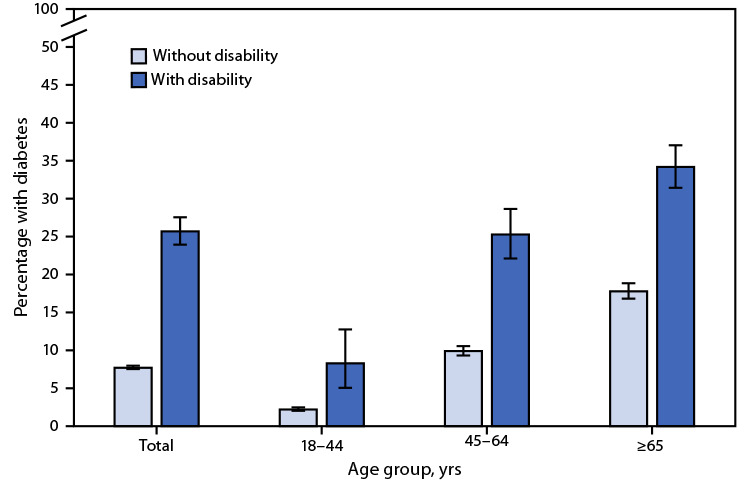
In 2020, 25.7% of adults aged ≥18 years with disability had diagnosed diabetes compared with 7.7% of those without disability. For each age group, those with disability were more likely to have diabetes: adults aged 18–44 years (8.3% versus 2.2%), 45–64 years (25.3% versus 9.9%), and ≥65 years (34.2% versus 17.8%). Regardless of disability status, the percentage of adults with diagnosed diabetes increased with age.

**Source:** National Health Interview Survey, 2020. https://www.cdc.gov/nchs/nhis.htm

For more information on this topic, CDC recommends the following link: https://www.cdc.gov/ncbddd/disabilityandhealth/features/disability-and-diabetes-prevention.html

